# *QuickStats:* Percentage* of Children and Teens Aged 6–17 Years Who Missed >10 Days of School in the Past 12 Months Because of Illness or Injury,^†^ by Serious Emotional or Behavioral Difficulties Status^§^ and Age Group — National Health Interview Survey, 2014–2016^¶^

**DOI:** 10.15585/mmwr.mm6644a13

**Published:** 2017-11-10

**Figure:**
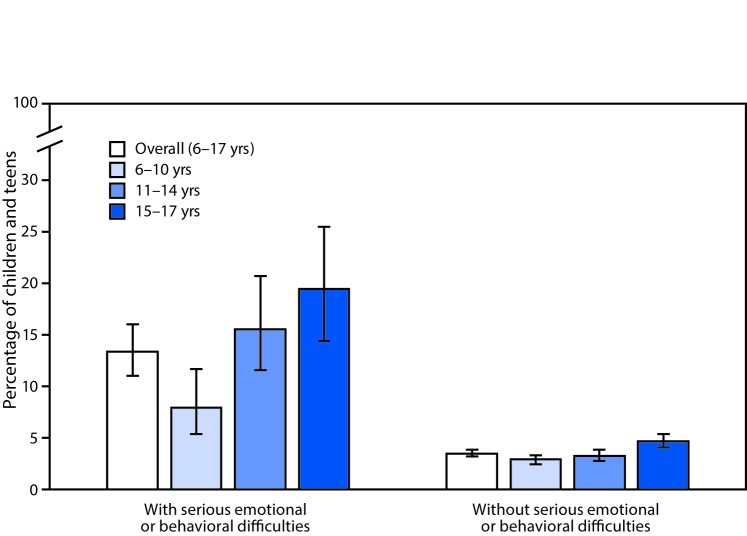
During 2014–2016, children aged 6–17 years whose parent or guardian indicated the child had serious emotional or behavioral difficulties (EBDs) were almost four times as likely to miss >10 days of school because of illness or injury compared with children without serious EBDs (13.4% compared with 3.5%). Among children with serious EBDs, those aged 6–10 years were less likely (8.0%) to miss >10 days of school compared with children aged 11–14 years (15.6%) and children aged 15–17 years (19.5%). Among children without serious EBDs those aged 15–17 years (4.7%) were more likely to miss >10 school days compared with children aged 6–10 years (3.0%) and children aged 11–14 years (3.3%).

**Source: **National Center for Health Statistics. National Health Interview Survey, 2014–2016. https://www.cdc.gov/nchs/nhis.htm.

